# The Cross-Link between Ferroptosis and Kidney Diseases

**DOI:** 10.1155/2021/6654887

**Published:** 2021-05-03

**Authors:** Jingyu Wang, Yi Liu, Yaqing Wang, Li Sun

**Affiliations:** ^1^Department of Nephrology, The First Affiliated Hospital of China Medical University, Shenyang, 110000 Liaoning Province, China; ^2^Department of Anesthesiology, Affiliated Hospital of North Sichuan Medical College, Nanchong, 637000 Sichuan Province, China

## Abstract

Acute and chronic kidney injuries result from structural dysfunction and metabolic disorders of the kidney in various etiologies, which significantly affect human survival and social wealth. Nephropathies are often accompanied by various forms of cell death and complex microenvironments. In recent decades, the study of kidney diseases and the traditional forms of cell death have improved. Nontraditional forms of cell death, represented by ferroptosis and necroptosis, have been discovered in the field of kidney diseases, which have reshuffled the role of traditional cell death in nephropathies. Although interactions between ferroptosis and acute kidney injury (AKI) have been continuously explored, studies on ferroptosis and chronic kidney disease (CKD) remain limited. Here, we have reviewed the therapeutic significance of ferroptosis in AKI and anticipated the curative potential of ferroptosis for CKD in the hope of providing insights into ferroptosis and CKD.

## 1. Introduction

Various cell death mechanisms participate in kidney diseases and are key targets for preventing tissue injury. However, a complex pathological process often comprises various interactions of cell death and pathological reactions, and intervention in just one or two of these may be difficult to achieve, especially in *in vivo* experiments. Nevertheless, the therapeutic potential of ferroptosis deserves recognition due to its enormous molecular network and cross-linking ability with multiple pathological reactions.

Ferroptosis, officially named in 2012, is characterized by intracellular iron accumulation and lipid peroxidation during cell death [1]. Recent studies have found that ferroptosis is involved in tumor development [2], neurodegenerative diseases [3], ischemia-reperfusion injury [4], and other pathological processes, and the targeted regulation of ferroptosis and its signaling pathways have achieved beneficial results. Studies on ferroptosis are considered to hold promise, especially for diseases involving other types of cell death without substantial advances.

Ferroptosis in kidney diseases has been studied primarily in acute kidney injury (AKI) and renal carcinoma, but this “stereotype” seems to be losing hold. Iron deposition in acute and chronic renal diseases is mainly the result of disturbed expression of iron metabolism-related proteins and high exposure of hemoglobin in the renal cortex [5, 6]. In patients with kidney diseases, lipid metabolism disorders result from multiple factors, such as obesity, hyperlipidemia, fatty acid intake disorders, fatty acid oxidation (FAO) disorder, and metabolic acidosis [7–9]. In this review, we elucidate the iron and lipid metabolism disorders encountered in AKI and chronic kidney disease (CKD) and describe their potential connection with ferroptosis.

## 2. The Characteristics of Ferroptosis

**Figure 1:**
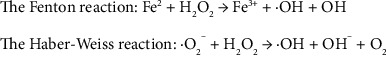
The Fenton reaction and Haber-Weiss reaction. Catalytic iron can react with oxygen to generate ROS such as hydroxyl radical and hydrogen peroxide, which are implicated in lipid peroxidation and tissue damage through the Fenton reaction. The Haber-Weiss reaction in cells can produce hydroxyl radical (^·^OH) from H_2_O_2_ and superoxide (^·^O^2-^), which is an important source of oxidative stress.

The characteristics of ferroptosis differ from those of other cell death types. The morphology of ferroptotic cells mainly manifests as mitochondrial changes, including reduced volume, increased membrane density, and decreased mitochondrial cristae, but the nucleus remains normal, with no chromatin agglutination [10]. Biochemically, intracellular glutathione (GSH) consumption and glutathione peroxidase 4 (GPX4) inactivation occur; thus, lipid peroxides cannot be metabolized by the reduction reaction catalyzed by GPX4. Besides, ferrous iron oxidizes lipids via the Fenton reaction and produces abundant reactive oxygen species (ROS) to further aggravate ferroptosis [11] (Figure 1).

Recently, researchers used glucose starvation to induce energy stress in immortalized mouse embryonic fibroblasts and investigate the energy stress effect on ferroptosis [12]. Interestingly, energy stress largely rescued cellular ferroptosis, in a process mechanistically linked to activating adenosine monophosphate-activated protein kinase (AMPK), promoting ATP production, and inhibiting polyunsaturated fatty acid (PUFA) synthesis [12, 13]. This reveals another feature of ferroptosis: cellular ferroptosis occurs without the loss of intracellular ATP [12–14]. As a vital sensor of intracellular energy metabolism, AMPK plays a crucial role in coordinating multiple metabolic pathways and balancing energy supply and demand [15]. AMPK activation could induce and inhibit ferroptosis, possibly because of its phosphorylation sites, environmental complexity, and activation level [16–18]. AMPK could also regulate mitochondrial homeostasis [19], and discovering mitochondria-independent mechanisms that inhibit ferroptosis has great implications. Additionally, AMPK activation causes the degradation of ferritin (Fer), accumulation of ROS, and activation of ferroptosis [20, 21]. A study suggested that AMPK is an upstream regulator of ferroptosis, and AMPK depletion sensitizes cells to ferroptosis [22]; however, due to insufficient detection of ferroptosis-related biomarkers and lack of mitochondrial phenotype support, the specific regulatory effect of AMPK on ferroptosis remains obscure. Assuming that the intracellular iron content and mitochondrial phenotype display an opposing trend to ferroptosis following AMPK deletion, despite lipid peroxidation occurrence, would this cell death still be considered ferroptosis? Is AMPK a regulator of ferroptosis? The answer is no in either case. Therefore, the rigor of the definition of ferroptosis is still worth discussing because of its great significance for related pathological diseases. As ferroptosis overlaps with autophagy, apoptosis, and oxidative stress [23–26], using biochemical ferroptosis biomarkers only to judge the occurrence or participation of ferroptosis is controversial because such a classification cannot exclude the pseudoferroptotic performance caused by other pathological factors.

## 3. The Main Regulatory Mechanism of Ferroptosis

### 3.1. Cystine/Glutamate Antiporter (System Xc^−^) and Ferroptosis (Figure 2)

System Xc^−^ is a widely occurring amino acid antiporter in the phospholipid bilayer, and its active subunit is SLC7A11 [27]. Glutamate (Glu) and cystine (Cys) enter and exit cells in equal proportions through system Xc^−^ [28]. Cys enters the cell to influence the synthesis of cysteine and GSH [1]. GSH activates GPX4 and significantly influences intracellular redox homeostasis maintenance. By inhibiting system Xc^−^ activity, sulfasalazine and sorafenib can interfere with Cys absorption and GSH synthesis, causing a decrease in GPX4 activity, a decline in cell antioxidant capacities and lipid ROS accumulation, and the gradual occurrence of oxidative damage and ferroptosis. Studies have reported that upregulating SLC7A11 could inhibit ferroptosis to cure diseases [29, 30]. Therefore, SLC7A11 is also considered a biomarker and executor of ferroptosis [29, 31].

### 3.2. GPX4 and Ferroptosis (Figure 2)

Among the family members of glutathione peroxidase (GPX), GPX4 is a crucial ferroptosis regulator. The activity of GPX4 is related to GSH content. When GSH is depleted, GPX4 activity is reduced or inactivated. As an antioxidant enzyme, GPX4 catalyzes the reduction of lipid peroxides and indirectly interferes with the Fenton reaction, which are important for maintaining the intracellular hydrogen peroxide (H_2_O_2_) content [32]. High H_2_O_2_ concentrations generate ROS and rapidly oxidize fatty acids (FAs) and arachidonic acid (AA) to produce lipotoxic substances. GPX4 also converts GSH into glutathione disulfide (GSSG) and reduces cytotoxic lipid hydroperoxides (L-OOH) to the corresponding alcohol (L-OH) [11], thereby resisting oxidative damage. Moreover, GSSG was reduced to GSH by GSH reductase and NADPH/H+ [33]. When GPX4 expression is downregulated, cells are sensitive to ferroptosis [34]. Conversely, ferroptosis was inhibited [34]. GPX4 and tumors are also linked by ferroptosis; although GPX4 inhibitors can induce ferroptosis in tumor cells, different tumor cells respond differently to GPX4 inhibitors. Therefore, researchers believe that there are other ferroptosis regulators in cells besides GPX4. In 2019, Bersuker et al. found that tumor cell resistance to the ferroptosis inducer RSL3 disappeared when ferroptosis suppressor protein 1 (FSP1) was knocked out, and the degree of cell ferroptosis increased significantly [35]. Coenzyme Q10 (CoQ) production was reduced by the mutation of the E156A site of FSP1, suggesting that inhibition of ferroptosis by FSP1 depends on CoQ oxidoreductase activity [35]. Accordingly, it was further demonstrated that FSP1 promotes lipid oxidation, which is related to CoQ oxidoreductase activity [36]. Therefore, molecular compounds that interfere with the activities of GPX4 and CoQ oxidoreductase could interfere with ferroptosis-related diseases.

### 3.3. Iron Metabolism, Oxidative Stress, Lipid Peroxidation, and Ferroptosis (Figure 3)

Cellular iron homeostasis is closely related to iron absorption, storage, circulation, and utilization [37]. The human gut absorbs approximately 1–2 mg of iron daily from the diet [38]. Dietary ferric iron is reduced to catalytically active ferrous iron with the help of cytochrome b on the brush border [39] and then enters intestinal epithelial cells under the regulation of the divalent metal transporter 1 (DMT1) [40]. In the cytosol, imported ferrous iron can either be stored in the ferritin heavy chain (Fth) or be regulated by ferroportin (FPN) to be exocytosed into the plasma. Exported ferrous ions are oxidized to ferric ions with the help of hephaestin and combined with transferrin (TRF) to start the iron cycle [41]. When hemolysis occurs or a large amount of iron is supplemented, the tissue iron concentration increases and even exceeds the TRF binding capacity, forming nontransferrin binding iron (NTBI). The liver, kidneys, and other organs are sensitive to iron, and their uptake and clearance of iron differ from those of the reticuloendothelial system, which may lead to tissue iron deposition and iron overload [41]. Additionally, a small quantity of ferrous iron is stored in the labile iron pool (LIP), which is used to maintain metabolism under physiological conditions [33]. During the iron cycle, NTBI and certain unstable ferrous irons can be easily oxidized and reduced through Fenton and Haber-Weiss reactions, and hydroxyl radicals (^·^OH) are produced, which may destroy macromolecules, such as lipids, proteins, and nucleic acids, resulting in oxidative damage (Figure 1). Ferrous iron can provide electrons through the Fenton reaction to promote lipid peroxidation and catalyze H_2_O_2_ decomposition, and the ^·^OH generated in this process accelerates intracellular ROS formation and induces ferroptosis. Certain enzymatic systems, including superoxide dismutase, catalase, and GPX, remove excess ROS to prevent tissue injury [33]. Oxidative stress occurs when ROS clearance and generation are imbalanced; then and there, large amounts of superoxide and peroxide induce ferrous iron release from iron-sulfur clusters, heme, and other iron-containing substances, causing increased ferrous iron concentration, which further aggravates oxidative stress through the Fenton reaction and forms a vicious cycle. Nuclear factor erythroid 2-related factor 2 (Nrf2) is a key transcription factor that regulates cellular oxidative stress and importantly influences intracellular redox homeostasis maintenance. When cells are stimulated by ROS, Nrf2 can be upregulated to relieve oxidative injury; Nrf2 activation then upregulates GPX4 expression [42], indicating that Nrf2 has an antiferroptotic effect and could be a regulatory factor of antiferroptosis. A recent study found that the inhibition of ferroptosis by Nrf2 is related to inhibition of antioxidant and iron metabolism gene transcription [43], which demonstrates a link between iron metabolism, oxidative stress, and ferroptosis, and that finding cross-linked pathways between the three could help to better neutralize iron-induced oxidative damage.

Autophagy is initiated to maintain homeostasis when cells are exposed to stressors, such as starvation and hypoxia. The ferritinophagy-induced increase in ferrous iron content triggers ferroptosis [44]. Catalytic iron, namely, ferrous iron, mediates lipid ROS production and is a key step in ferroptosis. The ^·^OH generated by the Haber-Weiss-catalyzed ferrous iron could extract a hydrogen ion from PUFAs to initiate lipid peroxidation, which promotes lipid peroxide accumulation and destroys the structure and function of the cytomembrane, causing ferroptosis. Lipid peroxidation is a free radical chain reaction, and its occurrence is related to the position and abundance of its substrates [45]. Free PUFAs are the substrates of lipid signal transduction mediators, but they must be esterified into membrane phospholipids to transmit ferroptotic signals [1]. When GPX4 activity decreases, its ability to scavenge lipid peroxides decreases. The PUFAs undergo continuous simultaneous oxidation. The above two sides collaborate to continuously accumulate lipid peroxides until ferroptosis occurs.

Interference with lipid peroxidation has become a target for ferroptosis and related pathological processes. Ferroptosis inhibitors, represented by ferrostatin-1, mainly inhibit ferroptosis by interfering with lipid peroxidation [46]. Researchers have found that AA and phosphatidylethanolamine (PE) are vital phospholipids that induce ferroptosis [1]. Acyl-CoA synthase long-chain family member 4 (ACSL4) and lysophosphatidylcholine acyltransferase 3 (LPCAT3) participate in the biosynthesis and remodeling of PE and affect the transmembrane properties of PUFAs. Downregulation of ACSL4 and LPCAT3 expression could affect the concentration of lipid peroxide substrates and inhibit ferroptosis. In the *GPX4* gene knockout AKI mouse model, thiazolidinedione could inhibit the antiferroptosis of ACSL4 and improve the survival rate of mice [47]. In summary, these results show that intervening in iron metabolism, oxidative stress, and lipid peroxidation can present therapeutic benefits to ferroptosis-related diseases.

### 3.4. P53 and Ferroptosis (Figure 2)

P53 is a tumor suppressor protein that plays an important role in cell starvation, hypoxia, oncogene activation, and other stress conditions [48], and its activation is related to the stress intensity [49]. In the nucleus, P53 promotes autophagy, whereas P53 in the cytoplasm blocks autophagy when stressed [50]. The contradictory effect of P53 on autophagy is related to posttranslational modifications [51]. P53 distribution in cells is expected to interfere with autophagy. Interestingly, P53 also has a contradictory effect on ferroptosis. P53-mediated promotion of ferroptosis is related to SLC7A11 inhibition and spermidine/spermine N1-acetyltransferase 1 (SAT1) and glutaminase 2 (GLS2) upregulation [52]. P53-mediated ferroptosis inhibition is related to decreased activity of dipeptidyl peptidase-4 (DPP4) and upregulation of cyclin-dependent kinase inhibitor 1A (CDKN1A) [52]. Unfortunately, the connection between P53 and the key regulators of ferroptosis remains obscure.

## 4. Ferroptosis and AKI

AKI is characterized by a sharp decline in renal function, and its incidence and prevalence continue to rise [53]. Recently, ferroptosis has been shown to participate in various pathological models of AKI, and *in vivo* and *in vitro* experiments have revealed that ferroptosis inhibitors are effective against kidney injury [54, 55]. Mice with *GPX4* deletion could spontaneously develop AKI [56], whereas GPX4 upregulation prevents AKI [57]. Due to the complex pathological process and diverse forms of cell death in AKI, the specific role of ferroptosis in AKI caused by different etiologies is unclear.

The regulation of iron metabolism can suppress inflammation, oxidative stress, and cell damage caused by iron overload and iron disorders. Heme oxygenase-1 (HO-1) influences enzymatic reactions that catalyze the decomposition of heme and promote the absorption of intracellular iron [58]. When HO-1 expression is suppressed or its enzymatic activity decreases, the intracellular ferrous iron content decreases [59]. Therefore, HO-1 is well known as a key regulator of iron metabolism. When AKI attacks, HO-1 induction occurs in the proximal tubule, whereas HO-1 deletion is sensitive to ferroptosis [60–62]. Many studies have demonstrated the protective effect of *HO*-*1* against AKI using the gene knockout and transgene [62, 63]. However, HO-1 overexpression—from toxin exposure—is harmful to the kidney and directly causes mitochondrial dysfunction; ROS is also actively involved in this process [64]. As the only iron export protein in mammals, FPN importantly affects plasma iron level maintenance. When the *FPN* gene was knocked out in AKI mice, the expression of GPX4 and Fth was upregulated and renal function was improved, which could have resulted from inhibited ferroptosis and Fth chelation of ferrous iron [65]. The regulation of iron by FPN is also dominated by hepcidin, which is a protective factor for AKI, and its antikidney injury is related to iron homeostasis restoration and inflammation inhibition [66, 67].

Catalytic iron plays a key role in promoting AKI progression [67]. The survival rate of AKI mice decreased significantly when the *Fth* gene was knocked out in proximal tubule cells [68]. This is consistent with the results of hospitalized patients, and plasma ferrous iron levels are positively correlated with AKI risk [69]. Although both the endogenous and exogenous iron chelation processes have been shown to alleviate tissue injury, using iron chelators in large quantities produces the opposite effect, and due to excessive chelation, the iron content that should maintain the physiological activities of cells is reduced, which can cause multiple organ failure [70–72]. In this section, we emphasize the significance of iron chelation in alleviating tissue damage, but we also stress the adverse effects of excessive chelation on tissues. Besides, due to the strong oxidizing properties of iron and its ability to generate ROS via the Fenton reaction, certain antioxidants and ROS scavengers have demonstrated protective effects [73–76]. Therefore, from the perspective of antioxidative injury, catalytic iron-mediated ROS removal is akin to iron chelation therapy. Eventually, whether this interferes with iron absorption or iron release may affect the expression of Fer. The key is how to chelate excess catalytic iron as much as possible while ensuring systematic iron homeostasis.

In the kidney, lipid accumulation—a durable response to renal insults—is an important factor that contributes to the aggravation of AKI, especially in AKI induced by ischemia-reperfusion injury [77–80]. Fatty acid oxidation (FAO) contributes to the main source of energy for renal cells, especially proximal tubular epithelial cells; however, when renal blood flow is disrupted, tissues thicken and harden due to local ischemia, hypoxia, and metabolic disorders. During ischemia, the balance between fatty acid intake and utilization by the kidneys is disrupted, and because of free fatty acids, it becomes more challenging for FAO to restore balance; thus, lipid accumulation occurs, while ROS accelerates lipid peroxidation [77]. Subsequently, during reperfusion, ROS levels increase, the inflammatory cascade is amplified, and kidney damage worsens. Carnitine palmitoyltransferase (CPT) 1 and 2 are the key rate-limiting enzymes of FAO [81, 82]. In the absence of CPT1, the continuous oxidation of long-chain fatty acids may cause metabolic disorders [81]. *CPT2* deficiency can cause fatty acid metabolism disorders and mitochondrial dysfunction and even induce AKI attacks [83, 84]. In summary, an intervention in FAO and lipid accumulation may help reduce lipotoxic substance sedimentation, restore mitochondrial energy metabolism, weaken the inflammatory cascade, and ameliorate AKI.

Oxidative injury mediated by lipid peroxidation is an unfavorable factor for AKI [85], while lipid peroxidation inhibition can alleviate kidney injury [86]. One month before Dixon proposed ferroptosis, some researchers found that deferoxamine (DFO), which was not listed as a ferroptosis inhibitor then, could inhibit lipid peroxidation and renal tubular epithelial cell necrosis to prevent renal failure [87]. Reducing the iron load eases lipid peroxidation [88]. Accordingly, iron overload aggravates lipid peroxidation and worsens kidney function [88]. In a prospective study, researchers found that acetaminophen could inhibit lipid peroxidation in children with cardiopulmonary bypass (CPB) but has no effect on the incidence of AKI [89]; the results of adult surgical patients are consistent with this [90]. In our opinion, this does not mean that inhibiting lipid peroxidation is not effective in preventing and treating AKI, because the CPB-mediated hemolysis places strain on the kidney, and the circulatory changes during CPB surgery affect renal blood flow and perfusion pressure, which far outweigh the corrective effect of acetaminophen on lipid peroxidation. Moreover, acetaminophen inhibits lipid peroxidation in the treatment of AKI [91]. Therefore, we reason that inhibiting lipid peroxidation remains a possible treatment strategy for AKI; however, the nonclassification of acetaminophen as a ferroptosis inhibitor, although it inhibits lipid peroxidation and prevents kidney injury, remains puzzling. Contrary to it being taken for granted, acetaminophen is regarded as a ferroptosis inducer due to its nephrotoxicity and GSH-depleting effects [92]. This may seem contradictory, but it also demonstrates that lipid peroxidation intervention alone is not enough basis for classifying agents as ferroptosis inhibitors or inducers and that the decisive factor remains the influence on key regulatory molecules of ferroptosis. Hence, classical ferroptosis inhibitors are capable of interfering with key molecules in the ferroptosis signaling pathway to resist AKI [46, 54, 93]. Moreover, lipid peroxidation could regulate apoptosis and autophagy through NF-*κ*B, PKC, MAPK, and other signaling molecules [25]. Thus, preventing lipid peroxidation has beneficial outcomes on apoptosis, autophagy, and ferroptosis.

## 5. Ferroptosis and CKD

CKD has become a major public health issue of global concern. By 2040, CKD will become one of the top five causes of patient death [94]. Most patients with CKD exhibit varying degrees of iron metabolism and lipid metabolism disorders. Theoretically, this provides favorable conditions for ferroptosis. Here, we elaborate on the association between renal iron deposition, lipid deposition, and ferroptosis in CKD, but renal anemia, iron deficiency, and hyperlipidemia encountered in plasma have not been elucidated.

In people with normal expression levels of iron regulatory proteins under physiological conditions, kidney iron accumulation does not occur [5]. However, in CKD, tubular iron accumulation may be caused by decreased FPN abundance and upregulated Fer and iron importers [5], thereby initiating Fenton-mediated oxidative damage [95–97]. Kidney iron deposition also occurs in different CKD syndromes [98–100]. Iron supplementation can seem a possible cause of renal iron accumulation. Indeed, exogenous iron supplementation can accelerate kidney iron accumulation [101, 102]. However, in the absence of exogenous iron supplementation, renal iron deposition occurs spontaneously in different types of CKD [99, 100, 103–105] and is related to the release of ischemia-, hypoxia-, and cytotoxicity-induced catalytic iron [106]. This suggests that CKD kidney iron accumulation initially induces ferroptosis. Therefore, the regulation of iron metabolism proteins is of great significance in restoring kidney iron metabolism and mitigating ferroptosis.

In unilateral ureteral obstruction- (UUO-) mediated CKD mice, HO-1 was distributed near the tubulointerstitium and glomerulus, peaked at 12 h after obstruction, and decreased one week after [107]. This indicates that HO-1 induction in CKD is a response to oxidative stress and inflammation. The cytoprotective effect of *HO*-*1* in the kidney was verified in subsequent gene knockout UUO mouse models [108]. HO-1 induction also inhibited the expression of TGF-*β*1 and proinflammatory molecules, including IFN-*γ* and IL-1*β*, and reversed kidney hypertrophy [109]. Poor repair after an injury is an important factor in kidney fibrosis deterioration. The expression of TGF-*β* is conducive to HO-1 induction, which is considered a therapeutic strategy against TGF-*β*-mediated kidney disease [110]. In recent years, the molecular mechanism of TGF-*β* and its downstream signaling pathways in fibrotic nephropathy have been widely recognized, but the response of HO-1 to TGF-*β* remains unclear and requires further investigation. Additionally, HO-1 was specifically expressed in glomeruli in streptozotocin-induced diabetic nephropathy (DN) models [111], and the induction of this enzyme could rescue podocyte apoptosis [112]. In DN mice orally treated with the antioxidant tempol, a similar HO-1-induced activity appeared [113], which indicates that HO-1 induction is beneficial for inhibiting oxidative stress and restoring redox balance. In conclusion, HO-1 induction can mitigate oxidative stress-induced histopathological changes, inflammation, and fibroblast proliferation.

Although iron regulatory proteins are being increasingly elucidated in recent years, drugs targeting HO-1 in clinical applications remain absent, which is undoubtedly the urgent expectation of nephropathy patients. The complicated genetic background and pathological processes have made it difficult to transform scientific research achievements into clinical applications in a short time, and this requires exploration.

Iron chelation is considered a treatment for renal fibrotic lesions [114]. The ferroptosis inhibitor, deferasirox, can mitigate renal fibrosis in CKD rats by inhibiting TGF-*β*1/Smad3, inflammation, and oxidative stress pathways [115]. DFO mitigates kidney fibrosis by alleviating iron metabolism and oxidative stress in UUO mice [116]. Besides, the inhibitory effect of DFO on inflammation was confirmed using *in vivo* experiments [117, 118]; however, this may not be the effect of DFO itself but a favorable reaction after oxidative stress inhibition. Moreover, an iron-restricted diet exerts a renal protective effect by inhibiting oxidative stress and aldosterone receptor signaling in animal models of CKD [119–121]. In conclusion, iron chelation treatment could delay the progression of CKD by inhibiting the proliferation of myofibroblasts through antifibrosis, anti-inflammation, and antioxidative stress mechanisms [114].

In the field of organ fibrosis, ferroptosis and liver fibrosis have been studied most. This may be because the liver is more prone to iron overload in pathological conditions [106]. Several studies have found that the ferroptosis-dependent mechanisms, including *GPX4* depletion, lipid peroxidation, ferritinophagy, and P53, are involved in liver and pulmonary fibrosis [122–125], and nontargeted agents regulating the ferroptosis signaling pathway also showed antifibrotic effects [126, 127]. Therefore, the perspective that ferroptosis is considered a therapeutic target for organ fibrosis has been gradually accepted [128, 129]. In a recent study involving ferroptosis and UUO-mediated renal fibrosis, researchers found that regulating the ferroptosis signaling pathway can alleviate kidney injury [130], but they failed to directly prove that ferroptosis is involved in renal fibrosis progression. Therefore, studies on ferroptosis and renal fibrosis remain to be conducted. Current studies have focused on the relationship between ferroptosis and oxidative stress, on which fibrosis depends, while less research has been conducted on ferroptosis and epithelial-mesenchymal transition (EMT), which still needs to be explored.

Hyperlipidemia and obesity are independent risk factors for CKD. Kidney lipid accumulation is thought to be the result of hyperlipidemia and/or obesity. Obesity and/or hyperlipidemia induces transport of adipose tissue lipids through the bloodstream to nonadipose tissues, a process known as ectopic lipid deposition [131]. However, CKD can spontaneously develop ectopic lipid deposition. In partial nephrectomy-induced CKD rats, researchers observed changes in lipid regulatory molecules in the residual kidney at 11 weeks postsurgery, suggesting that lipid deposition in the residual kidney is mainly the result of increased tubular lipid absorption and inhibition of fatty acid catabolism [132]. However, this study lacked time tracing due to the differing compensatory capacities of the residual kidney across time points postnephrectomy. Moreover, ectopic lipid deposition may be alleviated or aggravated by compensation or decompensation, respectively.

Lipid accumulation occurs in almost all kidney cells [133] and is positively correlated with the progression of CKD [134]; this is also consistent with epidemiological evidence [135–137], emphasizing the harmfulness of lipid accumulation in CKD progression. Besides, lipid deposition in the renal parenchyma can induce ROS release, promote lipid peroxidation, damage mitochondria and other organelles, and cause renal tubular and glomerular injury [8], indicating that oxidative stress plays an adverse role in lipid deposition [137, 138]. Therefore, regulating lipid metabolism and preventing ectopic lipid deposition are essential to alleviate tissue damage.

CD36 is a multifunctional transmembrane glycoprotein involved in lipid uptake by kidney cells. Especially in patients with DN, CD36 is highly expressed and positively correlated with ectopic lipid deposition [136, 139]. Sulfosuccinimidyl oleate, an antagonist of CD36, inhibits high glucose-mediated EMT in HK-2 cells, suggesting that inhibiting lipid absorption mitigates renal interstitial fibrosis [140]. CD36 is also cross-linked with inflammation and facilitates disease progression [139]. Therefore, it is not surprising that the regulation of the CD36 signaling pathway interferes with the production of lipotoxic substances and alleviates renal interstitial fibrosis [141]. Gene targeting technology has confirmed the key role of CD36 in ectopic lipid accumulation; mice with *CD36* gene deletion are less prone to renal function deterioration and related complications [139]. In conclusion, inhibition of lipid accumulation helps to alleviate lipotoxicity-mediated tissue damage, which may be due to the inhibition of proinflammatory, prooxidative, and pro-EMT effects of lipotoxic substances.

Direct studies regarding DN and ferroptosis were the first to be conducted in all types of CKD. The involvement of ferroptosis has been confirmed in streptozotocin-induced DN animal models [142]. To our knowledge, this is the first time that ferroptosis-specific mitochondrial changes in DN mice have been observed using transmission electron microscopy. Other researchers have also found ferroptosis-specific mitochondrial changes in the HK-2 cell line induced by high glucose, while fenofibrate regulates the ferroptosis signaling pathway to alleviate tissue damage [143]. In a recent study, typical features of ferroptosis were observed in kidney biopsy specimens of patients with DN [144]. This breakthrough will improve research on ferroptosis and DN, and studies will no longer be limited to the cellular and animal levels. However, it is unclear whether ferroptosis can be observed in patients with DN at different stages. Previous studies have focused on the role of podocytes in the pathogenesis of DN, but many unanswered questions remain. Tubule cells are closely related to iron metabolism and ferroptosis, and due to this feature, the role of tubule cells in DN may be gradually emphasized in future studies.

## 6. Conclusion

Taken together, ferroptosis cannot occur without two steps—iron accumulation and lipid peroxidation. The important role of ferroptosis in AKI has been discovered, and several small-molecule compounds targeting the ferroptosis pathway have shown their kidney injury-resisting ability. Although iron deposition and ectopic lipid sedimentation in CKD create favorable conditions for the occurrence of ferroptosis and oxidative stress boosts lipid peroxidation, experimental research involving ferroptosis and CKD induced by different pathological factors remains lacking. As the understanding of these pathways improves, interventions on ferroptosis and its signaling pathways may provide new treatment options for CKD. Moreover, the physiological role of ferroptosis remains unclarified, and methods should be developed to translate scientific research into clinical applications. The ferroptosis pathway is considerable, and its molecular network is complex. Not only does ferroptosis differ significantly from other forms of cell death, but its role and mechanism—compared with other forms of cell death—with CKD also remains unclear. The specific regulatory molecules involved in the ferroptosis signaling pathway in nephropathies require further clarification.

## Figures and Tables

**Figure 2:**
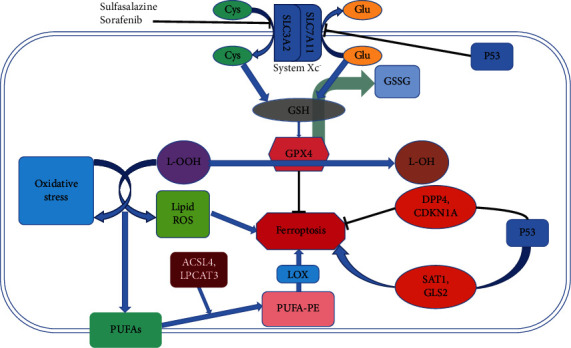
The main regulatory mechanism of ferroptosis. Abbreviations: Cys: cystine; Glu: glutamate; GSH: glutathione; GSSG: glutathione disulfide; GPX4: glutathione peroxidase 4; L-OOH: lipid hydroperoxides; L-OH: the alcohol of lipid hydroperoxides; PUFAs: polyunsaturated fatty acids; PE: phosphatidylethanolamine; LOX: lipoxygenase; DPP4: dipeptidyl peptidase-4; CDKN1A: cyclin-dependent kinase inhibitor 1A; SAT1: spermidine/spermine N1-acetyltransferase 1; GLS2: glutaminase 2.

**Figure 3:**
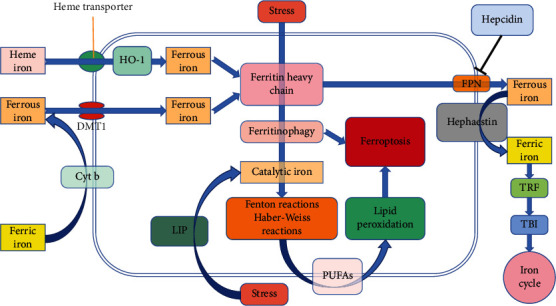
Partial cross-molecular mechanisms of iron metabolism and ferroptosis. Iron enters cells through different mechanisms: intracellular iron is either stored in Fth or regulated by FPN exocytosis into the plasma and oxidized to ferric iron, which then binds to TRF to start the iron cycle. The chelation of Fer to ferrous iron is limited. Under stress conditions, such as starvation and hypoxia, ferritinophagy is activated and catalytic iron is released in LIP. Catalytic iron can produce Fenton and Haber-Weiss reactions with ROS, affect the synthesis of PUFAs on the cytomembrane, and gradually induce lipid peroxidation and ferroptosis. Abbreviations: Cyt b: cytochrome b; DMT1: divalent metal transporter 1; HO-1: heme oxygenase-1; Fer: ferritin; Fth: ferritin heavy chain; LIP: labile iron pool; ROS: reactive oxygen species; PUFAs: polyunsaturated fatty acids; FPN: ferroportin; TRF: transferrin; TBI: transferrin binding iron.
